# Sense or sensibility—Ideological dilemmas in gamblers' notions of responsibilities for gambling problems

**DOI:** 10.3389/fpsyt.2022.953673

**Published:** 2022-07-25

**Authors:** Eva Samuelsson, Jenny Cisneros Örnberg

**Affiliations:** ^1^Department of Social Work, Stockholm University, Stockholm, Sweden; ^2^Department of Public Health Sciences, Stockholm University, Stockholm, Sweden

**Keywords:** gambling, individual responsibility, corporate social responsibility, interviews (qualitative), ideological dilemmas

## Abstract

The gambling market is a complex field of conflicting stakeholders and interests involving dimensions, such as economy, health, social inequality and morals. The division of responsibility between gamblers, the gambling industry and the regulating state for limiting the harmful effects of this activity, however, are unclear. The aim of this study was to explore how gamblers in the Swedish market attribute responsibility to various actors within the gambling field. Qualitative interviews were conducted with 37 gamblers experiencing extensive gambling problems. Based on a discourse analytical approach, five ideological dilemmas were identified, highlighting the tension between the, often contradictory, values that the participants need to relate to. On the individual level, the gamblers emphasize their own responsibility for their problem, thereby showing accountability in relation to themselves, their significant others and their peers as agents in recovery. On the corporate- and state levels however, the participants argue for a stronger public health approach, where the gambling companies should take further responsibility by living up to the legal regulations and where the state should ensure compliance and safeguard funding for treatment and research. The essential paradox between the individual responsibility discourse of self-regulation and the prevailing medical discourse of the gambler's incapacity for self-control signifies an impossible equation that imposes feelings of guilt and shame upon an individual who is concurrently considered as both responsible and incapable. In order to reduce harm, the gambling industry must be more proactive with coercive external control measures to fulfill the duty of care they claim to adhere to, and the regulating state must ensure its compliance.

## Introduction

The gambling field contains a variety of conflicting stakeholders and interests involving dimensions such as economy, health, social inequality and morals (1). The gambling industry and the regulating state have legal responsibilities to safeguard gamblers from problems, but also financial interests in gambling revenues. Value-conflicts are apparent for governments and the gambling industry in regard to the balancing act between considering substantial taxation revenue and profitability, on the one hand, and the economic and personal costs of gamblers and their significant others, on the other (2, 3). Ideas about responsibility permeate social life and form the basis for governance. The definition of responsibility includes, “individual and collective accountability through judgements of one's rational capacities, assessment of legal liabilities, and notions of moral blame” [(4) p.137]. However, key concepts, such as responsibility, tend to be vague and based on uncritical acceptance of its underlying assumptions (5). Due to the elastic (6) characteristics of responsibility, as a concept, it is seldom clear who should be held accountable and how. This is particularly evident in new markets where the division of responsibility has not yet been stabilized. In this paper we study how gamblers in the Swedish market attribute responsibility to various actors within the gambling field.

Perceptions of problematic behaviors and responsibilities are reflected in the values and interests of different actors possible in specific socioeconomic and political contexts (7). Historically, gambling has been viewed as an immoral vice as such, where no distinction was made between normal and pathological gambling (8). *The medical discourse* surrounding gambling problems as a pathological compulsion has been dominant since the 1980s. According to the classic medical model, “pathological” gamblers would not be framed as neither responsible nor guilty for their behavior, but rather as victims of a disease (9). During recent years, the view of responsibility throughout the medical discourse has widened and now regards individual gamblers as consumers who are responsible for their own decisions in the market (5). As a concept, *responsible gambling* (RG) was originally developed by the gambling industry as a response to community concerns of the harmful effects of gambling. A tripartite model was proposed where governments, industry, and individual gamblers would share responsibility in minimizing gambling-related harm (3). While governments hold the legislative and monitoring roles, the gambling industry would provide gamblers with safe products and relevant information to increase awareness of potential risks, thus enabling an informed consumer choice. The “burden of gambling responsibly” is, however, placed on individual gamblers in regard to considering potential consequences before deciding to engage in and throughout the duration of gambling activities [(3) p.567]. Orford (10) describes various dominant ways of thinking, in line with RG and supported by the gambling industry. The *ordinary business discourse* entails the promotion of gambling products as legitimate to market as any other commodity [visible not least in marketing and the desire to change terms from “gambling” to “gaming,” see (5)]. The *freedom to choose discourse* emphasizes the rights of citizens to make their own decisions about their lives and frames regulatory restrictions as an intrusion on individual integrity. Lastly, and most importantly, Orford (10) identifies the *individual responsibility discourse* where responsibility for protecting the health and wellbeing of individuals and their significant others is placed on respective consumers. They, thus, have an obligation to use these ordinary commodities in a sensible way, which is most clearly embodied within the concept of RG.

However, RG has been subject to substantial critique for being non-specific (6), unscientific (11), inadequate (12), and lacking credibility (13). RG has been promoted as harm reducing but also criticized for legitimizing deficient regulation and state-compliance toward the gambling industry (14). In addition, RG has been framed as problematic for placing responsibility for avoiding gambling harms on the individual rather than on the government or industry (15, 16). This reinforces the illusion of safe gambling as a possibility (17). The individual, as a consumer, is expected to control his/her behavior through rational, self-limiting and informed choices. This choice however presupposes capable and rational consumers, not inhibited by impaired control or pathological compulsion (5).

As an alternative, *a public health approach* has been suggested by several researchers, targeting universal population level approaches in favor of individual interventions. A public health approach considers the contribution of social, economic and demographic factors to gambling harm and involves measures, such as restrictions on gambling accessibility, regulations on advertising, age limits, and demands for identification. The focus is placed on population-wide gambling related harm, rather than on the level of the individual gambler. Therefore, responsibility falls mainly on the provider and the regulating state to protect the consumer from gambling harms (1, 12, 14, 18, 19).

The Swedish gambling market has recently been subject to substantial reform. With the Gambling Act (20), a new licensing system emphasizing consumer protection, was introduced. Starting on January 1st, 2019, foreign-based companies, previously attracting the Swedish market without a national license, can now apply for one. Although offline gambling such as land based casinos and EGMs still are exclusively run by the state owned gambling provider Svenska Spel, the new regulation has meant that about 90 new actors have a license on the Swedish market—mainly regarding online gambling and betting (21). ATG, the company owned by the national trotting and gallop organizations that previously had the legal monopoly of providing horse betting in Sweden, now offers online casino. In all, the availability of gambling has increased, but Svenska Spel and ATG still control a large share of the market. The law regulates the license holder's duty of care (“omsorgsplikt”) where social and health protection should be considered in order to safeguard consumers from excessive gambling and assist in gambling reduction, if needed. More specifically, the license holder should continuously monitor the gambling patterns of their customers, contact the gamblers if there are signs of excessive behaviors and inform them of available services for help. Provision of registration, gambling limits and self-exclusion through a national register covering all license holders, is mandatory (20). The extent to which the compliance of license holders will be controlled and sanctioned, however, depends on the resources and procedures of the gambling authority (22).

The discussion surrounding the reregulation of the Swedish gambling market has lasted several decades. Most licensed and unlicensed gambling companies within the Swedish market claim to conform to the legal obligations even before the new legislation was introduced. The gambling providers however have diverging views of what RG entails, emphasizing individual responsibility. Providing tools for the individual gamblers is, in many cases, considered enough (23). However, among the general public, awareness of gambling problems has increased due to the ubiquitous gambling advertising, including the appearance of celebrities and ordinary citizens revealing their life stories of destructive gambling habits in the media, as well as the political debate concerning the reregulation (24). Furthermore, Sweden has traditionally embraced a social model of addiction problems (25). Despite the increased medicalization of gambling problems that has taken place during recent years (26), substance use and gambling problems are still, to a large extent, handled by municipal social services rather than by regional health care (24, 27). The gambling field thereby contains a variety of interests where competing discourses of responsibilities for gambling harms are of topical importance. Gamblers' own perceptions of responsibility for gambling problems are, in this context, highly relevant.

To understand the complexity of gambling problems, the normative assumptions underlying the existing order of responsibility for gambling harm (2) and how they are entwined with social structures (28), must be scrutinized. The dimensions of responsibility for gambling harms includes the origin of the problem, its solutions and various actors, such as the individual gambler, the gambling industry, and the legislative and regulating state. Different forms of responsibilities are at play, at different levels; for example, the individual gambler's responsibility to him/herself as a self-governing subject as well as to his/her significant others, between the gambler and his/her peers in self-help groups and in the relationship between the citizen, the profit-driven gambling corporations and the regulating government (4). In this paper, we seek to show this diversity of responsibilities for gambling problems through accounts by gamblers themselves. The aim is to explore gamblers' perceptions of responsibilities for gambling problems in relation to dominant discourses of individual, corporate and state responsibilities for gambling problems. More specifically, how do gamblers with extensive negative consequences from their gambling attribute responsibility to different actors in the gambling field and what are the assumptions underlying their potentially competing notions of responsibilities? To conclude, possible implications of the dominant discourses regarding attributions of responsibilities and their relationship, are discussed.

## Materials and methods

### Data collection

Participants for this study were recruited through internet and social media advertising, as well as from available treatment options and self-help groups in different parts of Sweden. In-depth, semi-structured interviews were conducted with 37 gamblers during 2018 and 2019, in the midst of the reregulation of the Swedish gambling market. The interviews (mean 63 minutes, SD 14) were conducted over the telephone (*N* = 32) or, if preferred by the interviewee, face-to-face (*N* = 5). In addition to open ended questions about the participants' notions of responsibility in regard to gambling harm, the interviews covered topics, such as the nature and course of gambling problems, as well as potential views on and experiences of help-seeking and RG measures. After having received the participants' informed consent, the interviews were audio recorded. All interviewees were given fictitious names, which are stated in the Results section alongside gender, age range and main form of problematic gambling.

### Participants

As displayed below, in Table 1, the majority of participants were men, most of whom were employed and living with a partner and children. The median age was 38 (SD 10) and their social situation varied to the extent that some of the interviewees described themselves as homeless and isolated, while others would identify as successful business managers with strong social networks. The participants came from both urban and rural areas, predominantly from the middle or southern parts of Sweden. About one fourth (nine out of 37) of the interviewees had foreign backgrounds (born abroad or having at least one immigrant parent). All participants expressed dealing with previous or ongoing issues of severe gambling problems with socially-, economically-, judicially- and health-related consequences [NODS PERC mean 3.8 of 4, (29)]. While the majority reported 6–15 years of gambling problems (median 9 years, SD 9), six of them noted ongoing issues. More than half of the interviewees had refrained from gambling for only 1–6 months at the time of the interview (median 4 months, SD 23). Out of the 37 participants, 9 (24%) described simultaneous substance use problems. As shown in Table 2, the main problematic forms of gambling were online casinos and sports betting, which reflects the increase in gambling problems among Swedish online gamblers during recent years (30).

**Table 1:** Description of participants.

	***N***	**%**
Gender	
Women	7	19
Men	30	81
Age category	
24–30	7	19
31–40	16	43
41–50	7	19
51–65	7	19
Employment status	
Employed	21	57
Student	5	16
Unemployed, retired, on sick leave or social allowance	11	27
Civil status	
Single without children	11	30
Single with children	5	14
Partner without children	6	16
Partner with children	15	40
Years with gambling problems	
0–5 years	8	22
6–15 years	19	51
16–45 years	10	27
Months free from gambling at the time of the interview	
0 months	6	16
1–6 months	19	51
7–19 months	6	16
20–100 months	6	16
Recruitment	
Self-help groups	14	38
Treatment options	14	38
Social media and internet ads	9	24
Geographical area	
Northern Sweden	4	11
Middle of Sweden	21	57
Southern Sweden	12	32
Area^a^	
Large city	14	38
Mid-size town	13	35
Small town	10	27

N = 37.

^a^Based on number of inhabitants in the municipality. Large city < 500,000, Mid-size town 50,000–499,999, Small town > 49,999.

**Table 2:** Participants with listed pseudonym, gender, age range and main problematic gambling form.

**Pseudonym**	**Gender**	**Age range**	**Main problematic gambling form(s)**
Alexander	Man	41–50	Offline and online casino
Amir	Man	41–50	Online casino, sports betting
Anders	Man	51–65	Offline sports betting, number games, EGMs
Anna	Woman	51–65	Online casino
Axel	Man	31–40	Sports betting, poker
Bengt	Man	51–65	Offline and online casino
David	Man	24–30	Poker, online casino, sports betting
Emil	Man	41–50	Horse betting
Erik	Man	24–30	Online skins gambling
Fredrik	Man	31–40	Offline horse and sports betting
Gustav	Man	24–30	Online casino, horse and sports betting, EGMs
Hans	Man	31–40	Online casino, sports betting, EGMs
Ivan	Man	41–50	Offline casino and card games
Jan	Man	31–40	Online sports betting
Jemal	Man	41–50	Offline casino and sports betting
John	Man	31–40	Online poker, sports betting
Karin	Woman	51–65	Online casino
Karl	Man	24–30	Online sports betting
Katarina	Woman	31–40	Online casino, EGMs
Kristina	Woman	31–40	Online casino
Lars	Man	51–65	Online casino, horse betting, lotteries
Lena	Woman	41–50	Online casino
Magnus	Man	41–50	Online poker, sports betting
Markus	Man	31–40	State offline casino
Nils	Man	31–40	Online casino, sports betting, EGMs
Olof	Man	31–40	Online and offline casino, poker, EGMs
Omar	Man	31–40	Offline and online casino, EGMs
Oskar	Man	31–40	Online poker, sports betting
Per	Man	31–40	Online casino, poker, horse and sports betting
Rickard	Man	51–65	Online poker and casino, number games
Robert	Man	31–40	Offline and online horse and sports betting
Robin	Man	24–30	Online casino
Sara	Woman	31–40	Online and offline casino, EGMs
Sofia	Woman	24–30	Online casino
Stefan	Man	24–30	Online casino
Thomas	Man	51–65	Online casino
Viktor	Man	31–40	Online sports betting

### Coding

The interview recordings were first transcribed verbatim. The transcriptions were read repeatedly while listening to the audio tapes in order to capture the intent and context of the accounts as told by the interviewees. Approaching a material always depends on theory and prior assumptions (31), which in this case consisted of the available discourses of apprehending gambling responsibility as described by Orford (10), Reith (5, 32), and Livingstone and Rintoul (12) within the introduction. The coding and analytical procedure can best be described as a reflexive (31) or iterative circular process between the concepts and the interview material (33). All accounts covering the topic of responsibilities for gambling harm were first extracted in the software program NVivo and coded to specify individual-, corporate-, and state-level responsibilities. The data was then coded according to more abstract themes emergent in the participants' ways of talking about gambling responsibilities, related to core values, such as capacity, moral obligation, blame, accountability, legal liability, and rationality.

### Analytical approach

The lived ideology that shapes our ideals and practices within a specific context is seldom coherent, but rather filled with contradictions (34). Different ways of talking about an issue does not occur spontaneously or independently. Instead, they are shaped by historical, social and argumentative positioning (7). Throughout the gamblers' stories, various competing arguments regarding capacity, responsibility, freedom, moral and rationality, emerge. A discourse analytical approach (35) was, therefore, applied in order to highlight the contradictions in the gamblers' argumentations (7). The concept of ideological dilemmas (34) was utilized to shed light on the characteristics of everyday notions of gambling responsibilities, as well as the assumptions that form the basis for different reasonings. Ideological dilemmas are contradictory themes that evolve around a dialogue of opposing ideals. The dilemmas constitute the building blocks of our thoughts on a matter—notions throughout society that also shape our self-identity, moral assessments, decision-making and actions are often taken for granted. These dilemmas also highlight the opposing values, which gamblers are subject to during their active gambling episodes. In addition, gamblers actively have to navigate the negative consequences of their actions after having quit gambling and construct explanations for those occurrences. This article, thus, contributes to building an understanding of the tensions and the difficult balancing act that gamblers are confronted with in regard to responsibility and accountability.

## Results

**Figure 1:**
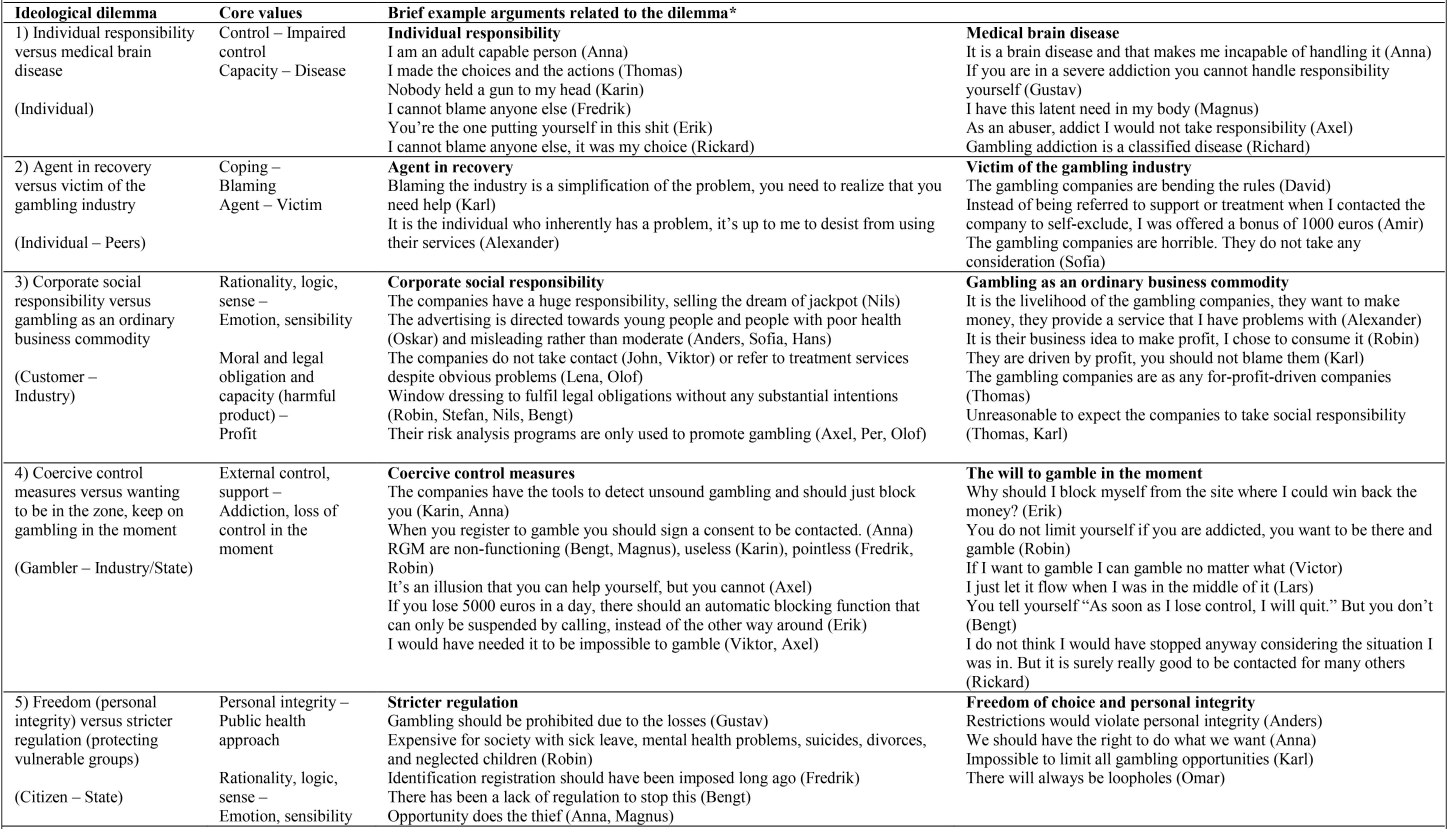
Ideological dilemmas, core values and brief example arguments *Quotes are abbreviated to fit the table while keeping its primary aim.

Five different ideological dilemmas were identified highlighting the tensions between responsibilities for gambling problems that participants struggle with when trying to make sense of themselves, their gambling problems, the gambling market and society. Opposing values, which are not mutually exclusive, but interconnected, permeate the ideological dilemmas, accentuating the complexity in which the gamblers relate to responsibilities on different levels. The five identified dilemmas, various accounts displaying them and the arguments used are clarified in Figure 1 and below.

### Individual responsibility vs. medical brain disease

The first dilemma is centered around *individual responsibility*, which presupposes free will and capacity to control one's gambling, vs. having a *medical brain disease* where incapacity and impaired control is inherent. On the one hand, the gamblers are, according to the discourse of *individual responsibility*, required to self-regulate and control their gambling habits in various ways. The basic assumption is that they have acted based on their own free will, meaning it is the individual who has made the choices and decisions (“nobody held a gun to my head,” Karin) and that they are mature and capable (Alexander). On the other hand, based on a *medical discourse*, those who suffer from the brain disease are incapable of self-regulating their behavior. When the gamblers are asked how they perceive responsibility for gambling problems, their answers appear according to a distinct rhetorical structure as displayed in Anna's account below.

*Anna: Well, the responsibility is mine really. I am an adult, I am not legally incompetent. But the problem is that it is a brain disease. And that makes me incapable of handling it. (Woman, 51–65 years, online casino)*

Anna, thus, begins to place the primary responsibility on herself, and thereafter makes use of the medical discourse to disclaim her accountability. Limit-setting, self-exclusion and other RG measures are encouraged, but these are not perceived by participants as applicable when sick. There is, thus, a substantial difference between those who can (responsible gamblers) and those who cannot (disordered/pathological gamblers) control their gambling behavior—an argument where the responsible gambling discourse and the medical discourse overlap. However, being responsible, in accordance with the individual responsibility discourse, and concurrently sick and incapable, in accordance with the medical discourse, constitutes a dilemma, or an impossible equation, that causes shame and guilt among the participants. For example, Magnus below strives to be a competent person. He first claims accountability for his actions, but thereafter describes this latent need that renders him incapable of controlling his behavior. This divergence, along with the shame of letting himself be deceived by the appealing offers presented by gambling companies, give him strong feelings of self-recrimination.

*Magnus: First and foremost I see it as my own fault. That I am…well that I have this need. I would want to say that I am able to quit but it is always with me latent in my body. They are making it too easy for a gambling addict to continue gamble. And they want my money, they want to increase their profit. /…/ They don't take responsibility, but they take money. Because they want to make money, and preferably more money. And I am stupid enough to buy it. /…/ I have an inculcated habit of wanting to be competent. I want to perform well, both in private and professionally. I am decently successful professionally. Then it does not look good to come home and have gambled for ten thousand euros. That is not success. It is embarrassing and shameful. (Man, 41–50 years, online poker and sports betting)*

To navigate these opposing expectations of control and capacity is difficult for the participants. But individual responsibilization also serves another purpose, which becomes evident in the second dilemma.

### Agent of recovery vs. victim of the gambling industry

The second ideological dilemma emerging from the gamblers' approaches toward responsibility is related to the position as an *agent* vs. a *victim*. On the one hand, the gambling industry is framed as an unscrupulous exploiter of vulnerable people, and that this activity should be prohibited considering its consequential harms. As expressed by Gustav, “in a perfect world, gambling would be totally forbidden.” Sofia feels resentment toward the industry:

*Sofia: The gambling companies are horrible. They do not take any consideration. These companies aim to bleed the life out of people who have problems. Because it is the ones who cannot handle it that they make money off. The ones of us who slip into the pitfalls and gamble everything we have. They build the dream that we will get money to become financially independent. /…/ It's very treacherous. (Woman, 24–30 years, online casino)*

But on the other hand, the participants argue that, based on a rational stance, one cannot expect anything else from a for-profit-driven industry and, therefore, every man for himself has to learn how to cope. Taking responsibility for problems imposed on oneself and one's significant others, serves the purpose of portraying yourself as an active agent within a recovery process. Here, the only reasonable stance is to avoid placing blame on someone else for your precarious situation. Taking accountability for your actions becomes a way to show yourself, your family and your peers that steps are being taken toward change. Placing responsibility on the gambling industry or the regulating state is not considered helpful in a process for active change. With this reasoning, the participants differentiate between sense and sensibility.

*Alexander: That's something that I have discussed with many people since I started seeking help. That I as an individual have to learn to deal with my gambling. And, rather, not gamble at all since I cannot handle it. I cannot blame the gambling companies. They provide a service that many might be able to handle. /…/ If half of the Swedish population had a problem with this, then there would probably be a law in the parliament stating that they cannot market it or anything. But that's not the case, so I have to learn to deal with it somehow. It's all around me and it's up to me to desist from using their services. (Man, 41–50 years, offline and online casino)*

The only reasonable approach is thus to take active responsibility as an agent and learn how to handle it, rather than considering yourself a victim of the vicious race of the gambling companies. To be able to present any kind of critique vis-a-vis the industry or the state for their lack of efforts in preventing gambling harm throughout society, the participants must first admit their own guilt, wrongdoings and accountability.

### Corporate social responsibility vs. gambling as an ordinary commodity

The third ideological dilemma apparent throughout the participants' narratives is related to the capacity, legal and moral obligation of the gambling companies, on the one hand, and gambling as an *ordinary business commodity*, on the other. The gamblers express strong criticism, based on their lived experiences extensive and destructive gambling over the course of many years. Within their accounts, the lack of corporate social responsibility is comprehensive. The gamblers are also very aware of the legal obligations for gambling companies to follow duty of care by monitoring the customers' habits and to proactively act against excessive gambling patterns. The gamblers' narratives contain numerous examples of situations where gambling companies ought to have reacted to obvious destructive gambling habits, however, neglected to do so.

*Victor: You do not have to be a rocket scientist at the gambling companies to see that I have a huge problem with my gambling. I withdraw money and five seconds later ask them to hurry up the withdrawal. When it has reached my account, I have deposited it straight away again. /…/ There are numerous patterns they could have detected, they know I have a gambling problem. /…/ It is obvious for them that I, and many others, have problems with it. But instead they are encouraging it. (Man, 31–40 years, online sports betting)*

These experiences occurred both before and after the new Swedish legislation came into force, which might have increased the participants' expectations of the actions and compliance of companies and the supervision of the state. Considering the extensive societal harms of gambling, and the fact that a harmful product is being sold, the participants argue that the industry not only has a legal, but also a moral obligation to safeguard their customers. Also, in this case, the division between sense and sensibility constitutes a watershed.

*Robin: If I should reason with logic, they are companies and their business idea is to make profit. There is nothing strange with that. This is a product that I am consuming… I chose to consume it. No matter how much I want to blame them… they are disgusting when they are sending e-mails tempting with bonus offers. But I am making an active choice when I am gambling. /…/ But emotionally, I hate the whole industry and it should be illegal. People commit suicide. It destroys families. /…/ It is really expensive for society with all the sick leave, mental health problems, suicides, divorces, children in trouble. (Man, 24–30 years, online casino)*

The participants further reject the whole concept of corporate social responsibility with reference to their own experiences of discrepancy between what companies are claiming and actually doing.

*Alexander: It is their livelihood, why would they contribute to people gambling less? They want to make more money. /…/ They [the gambling companies] provide a service that I have a problem with. It is the same thing as maybe when people have eating disorders. Just that there are companies that provide food and somebody gets the idea to eat way too much of it. It's not their [the companies] fault, but the individual who inherently has a problem with that. (Man, 41–50 years, offline and online casino)*

On the other hand, considering the gambling industry as profit-driven, some gamblers admit, with resignation, that they do not expect the industry to show any legal or moral concerns. From a somewhat cynical or ostensible reasoning, the participants argue that anticipating social responsibility from these companies would be irrational. The consumer protection policy claims throughout the gambling industry are described as “hypocritical” (Nils, Bengt) and as “empty words, lip service” (Stefan), set up solely to fulfill legal obligations without any substantial intent or control. Since gambling is alike any other commodity (cf. Alexander's analogy to eating disorders above) and corporate social responsibility is viewed only as a false front, it is up to the individual to handle the problem.

*Karl: I do not think you can blame the gambling companies. Sure, they see that you place large bets over, and over again, but they are driven by profit. I don't think you should blame them. /…/ And sure, you can be critical and say that the companies should warn you and call you, but at the same time they want to make money. It's their business concept./…/ I understand that. If you are a rational human being, you understand that. (Man, 24–30 years, online sports betting)*

### External control vs. the will to gamble in the moment

The fourth ideological dilemma present in the gamblers' narratives is related to gambling availability and is concentrated on *the need for external control and concern* vs. *will and denial in the moment*. On the one hand, interviewees advocate for more coercive measures to limit the extensive harms they have experienced as a result of their gambling. According to this reasoning, gambling providers should actively block gamblers who have displayed obvious destructive patterns and directly offer support and treatment ideally provided by people with own experiences of gambling problems. Gambling companies are, thus, expected to take a more proactive role in excluding gamblers, something that is also postulated within the new regulation. Again, in accordance with the medical discourse, the participants believe that it is not reasonable to expect gamblers to self-regulate in the moment, since the focus is impaired control, which is why external forces are needed.

*Erik: You do not consider it before it is too late. When you are having the most fun you do not consider that you could get carried away, only once when you have lost it all. And there is always the desire to win back the money. And then it is even harder. Why should I block myself from the site where I can win back the money? I think it's really problematic to say that the person struggling with addiction should do it all by himself. (Man, 24–30 years, online skins gambling)*

On the other hand, the participants admit that if they had been excluded during an active gambling episode it would have probably have caused them a substantial amount of frustration. What they strive for in the moment is nothing else than to continue gambling. Victor recounts how he has strained himself to self-regulate, in various ways, and after setting a limit getting so frustrated with that limit that it triggered him to gamble in even higher amounts on another gambling site. His account displays the duality between the unfeasibility of limiting all infinite opportunities to gamble, on a global level, and the need to restrict all gambling opportunities, to be able to refrain, on the individual level.

*Victor: Since I gambled with larger sums, the limits I set were unreasonably high. I tried to set a lower limit on 50 euros. But the anxiety and anger towards that limit took over. Instead I went to another site and gambled for five times that sum. It did not work for me but just triggered me in another direction. /…/ These limits, you can always… If I want to gamble I can gamble no matter what. /…/ You can make it harder but not impossible. I would have needed it to be impossible. (Man, 31–40 years, online sports betting)*

Regarding the idea of being contacted by a gambling provider out of concern, the participants acknowledge that denial would have probably been the first reaction. But if such duty of care had been performed, Bengt believes that it might have contributed to him having changed his gambling behavior at an earlier stage. Lars was actually contacted by the Svenska Spel state gambling provider after having gambled excessively. His first reaction reflected a sense of being caught in the act and exposed, but in retrospect he appreciates it.

*Lars: Overwhelmed by a horrible shame. But we are experts at lying and the first thing that comes out of your mouth when you're an abuser, an addict, is a lie. They told me where to get help and support. And that is very good. (Man, 51–65 years, online casino)*

In the moment, caught in the game, the gamblers do not perceive various RG measures as relevant. To react and contact a gambler or execute an automatic blocking function, as a state or private gambling provider, would, thus, violate personal freedom, but also, have the capacity to simultaneously limit gambling harms. Since “opportunity makes a thief,” as expressed by Anna and Magnus below, availability restrictions and external coercive measures are needed.

*Magnus: If there is no possibility for me to gamble I will not gamble. It is the opportunity that does it…If it would be more regulated and limited that would prevent it. And that would be good for a person like me, but above all for new gamblers. Making it harder, demanding registration and so forth. (Man, 41–50 years, online poker and sports betting)*

### Stricter regulation vs. freedom and personal integrity

The role of the state in relation to the gambler, as a citizen, is pivotal throughout the fifth ideological dilemma. Since the Swedish state should be regulating and supervising the gambling market, while simultaneously operating the largest gambling provider, Svenska Spel, this role is ambiguous. On the one hand, the interviewees argue for a stronger public health approach. Generally, they feel positively toward the new regulations, but are disappointed in the state concerning that too little has been done too late to limit gambling harms, for example by restricting gambling advertisements.

*Oskar: There is no country in the world that allows this much gambling advertisement. It is sick, because it is directed towards young people /…/ and people with poor health. People who are lost or have a tendency towards escaping. /…/ I find the responsibility really immature from the gambling companies and the state in general (Man, 31–40 years, online poker and sports betting)*

The role of the state should also be to ascertain earmarked money for research and available treatment for those in need. Axel is upset about the lack of opportunities for treatment for people experiencing gambling problems, and that the costs for such treatment are assumed by municipalities instead of the state-owned gambling company, which experiences a large turnover each year. When compared to the alcohol market, which is relatively strictly regulated in Sweden, the participants argue that the state has been too passive and complacent toward private companies' and their own state gambling provider's aspirations for revenue. Considering the extensive harms caused by gambling to individuals, concerned significant others and society at large, stricter regulation is advocated for by the interviewees. This includes the supervision of loan markets and banking transfers. In some cases, prohibition is also advocated for, to protect vulnerable groups throughout society.

*Bengt: It is not just that I am blaming others, but I feel that there has been a lack of regulation to stop this. /…/ These double standards. It is strict that we cannot drink. We have the alcohol retailing monopoly. But when it comes to gambling there has been a green light. There has been so much illegal gambling with these machines and the secret rooms behind closed doors… /…/ My life would have been totally different today… (Man, 51–65 years, offline and online casino)*

On the other hand, measures that overly interfere with personal freedom would violate individual integrity. In line with the *personal freedom discourse*, and based on a neoliberal stance, the participants believe that prohibition or overly extensive restrictions are impossible.

*Anders: You cannot prohibit [gambling]. You cannot prohibit people from going to the alcohol retail store and you would not be able to prohibit people from going to the betting shop either. It brings revenue to the state, both alcohol and gambling. It's difficult to come up with a solution. /…/ If you cannot do it with alcohol… I would not know how to do it. That would restrict personal integrity. (Man, 51–65 years, offline sports betting, number games and EGMs)*

According to this argument, the state should not restrict freedom of choice. The interviewees further contended the unfeasibility of limiting all gambling options. Legal and illegal gambling opportunities are omnipresent, and as expressed by Omar, there will always be loopholes, irrespective of strict regulations.

*Omar: Most people cannot gamble. You need to show your ID. But people here in my neighborhood know the owners or the staff, and then it does not matter. They are gambling with someone else's personal identity number. /…/ There is this guy who sells personal identity numbers for 5 euros to make money. /…/ Svenska Spel has some control but not 100% control. (Man, 31–40 years, offline and online casino, EGMs)*

## Discussion

In times of increasing demands for individual responsibility (2), and within a context of the reregulation of the Swedish gambling market (36), the aim of this study was to explore gamblers' notions of responsibilities for gambling problems. Through in-depth interviews with gamblers and an analysis of the coherent ways of talking about gambling responsibilities, this study has shown diverse ways of comprehending responsibility on various levels. The analysis focused on disentangling the contradictory arguments apparent within the participants' approaches toward responsibility, constituting ideological dilemmas (34).

The first identified ideological dilemma—individual responsibility vs. medical brain disease—was centered around notions of capacity and control. In line with the dominating responsible gambling discourse of individual responsibility, the gamblers are required to regulate their gambling in various ways. But inherent to the prevailing medical discourse and the gambling disorder diagnosis, the “pathological” gambler is incapable of self-control. The brain disease model provides an accessible explanation as to how the gambler should relate to the problem (26), potentially helping to ease the burden of guilt and shame. By confirming to the sick role (37) and repeatedly framing gambling problems as a chronic disease, many of the interviewees struggled to present a legitimate excuse for their behaviors. However, since the notion of incapacity collides with the prevalent ideal of self-regulation, control and rationality, the constant failures of controlling their destructive gambling habits increase the interviewees' self-recrimination and guilt. When gamblers cannot live up to expectations regarding norms of self-control, self-realization, responsibility and rationality, permeating consumer society (32), he or she is pathologized or moralized. The emphasis on individual responsibility among gamblers was recently found in respective Canadian (38) and Australian (39) studies, where values, such as self-control, making the right decisions and enjoying the game were essentially identical to the core messages presented throughout the responsible gambling discourse. The strong focus on the individual gambler's responsibility to self-regulate is also apparent in the field of gambling research, where less attention has been paid to other stakeholders' roles and efforts to reduce harm (40) and critical perspectives of the RG paradigm at large have been lacking (41). When gamblers internalize messages from the individual responsibility discourse, increased stigma, self-blame and health disparities are to be expected (42–44). This self-blame can been understood in relation to notions of self-responsibility, self-control and freedom of choice permeating todays' neoliberal society (38), which naturally also influences our views of gambling and addiction. When gamblers fail to self-regulate their behavior, they are labeled pathological and irresponsible, resulting in substantial shame and self-stigma.

The second dilemma visualizes the tension between presenting the gambling companies as unscrupulous exploiters which implies a position of the gambler as a victim. By contrast, the participants, as self-governing subjects, have to instead take responsibility for their actions and choices in order to prove this ability to themselves, their significant others and their peers, whether in self-help and treatment settings, or during the interview. On this level, individual responsibility is a prerequisite and component of framing oneself as an individual taking accountability within a process of recovery. This discourse of individual responsibility, as described by Orford (10), is so strongly positioned within the interviewees' notions that it constitutes a prerequisite for reasoning with responsibility and accountability and for formulating any kind of critique against the state or industry. Blaming your gambling problems on someone else is not considered as helpful as an agent in recovery, but the individual must learn how to handle his/her problem in order to be able to cope. The tendency to take responsibility, as a coping strategy part of a recovery process, has been found previously (45). Individual accountability is also encouraged throughout the self-help movement and within the cognitive behavioral oriented treatment programs often offered (2). As opposed to being in denial, seeking help and holding yourself accountable for the problems caused is part of the journey toward making positive life changes and putting problems behind you. In this sense, framing individual responsibility through self-blame serves a purpose as an active agent in recovery.

The third dilemma is related to the gamblers' expectations of the industry—stronger corporate social responsibility vs. gambling as an ordinary commodity. According to the participants, gambling companies have a legal and moral obligation, as well as the capacity to exert duty of care. However, the lack of experience among the participant of such concern leaves some of them with the argumentative option to frame gambling as an ordinary business commodity (10). If gambling is framed as any product such as, for example food that the individual chooses to consume excessively, it is up to the individual to desist from such activities (32). Framing oneself as primarily responsible for the gambling problems before being able to critique the gambling industry and the state regulator was one rhetorical argumentative structure apparent throughout the participants' accounts. Another was to differentiate between sense and sensibility. From an emotional point of view, the interviewees expressed that gambling should be prohibited or at least more strictly regulated, considering the extensive harms caused by the product to individuals, families and societies. From a logical point of view however, they exhibit a quite ostensible and cynical stance when expressing that one cannot expect gambling providers, as profit-driven companies, to take responsibility for their customers. Therefore, based on their lived experiences, the gamblers dismiss the whole idea of corporate social responsibility—a complete rejection of the intentions and efforts made by the gambling industry. The gambling companies are portrayed as immoral and deceitful and the state's endeavor to limit gambling harms is described as insufficient. The confidence of the gambling industry in the general public and among gamblers, in general, is continuously low (46) despite the new regulations. Almost two thirds (64 percent) of Swedish people who have gambled during the last 12 months believe the gambling industry does not take enough societal responsibility (47). To improve their reputation and their customers' faith in their conduct, the gambling companies need to live up to the postulations they claim to adhere to.

The fourth dilemma involves the tensions between the need for external control and the ambition to continue gambling in the moment. Where Goffman (48) described gambling as an activity on the fateful threshold between retaining and losing control over the consequences of one's actions, Schüll (16) characterizes contemporary machine gambling as a pursuit of “getting into the zone.” The zone is a state of isolation from the insecurities in the outside world, where time, space and social identity, together with the obvious risk attached to spending money on gambling, are suspended. The gambler's motive for continuing to gamble, rather than winning, is encouraged by the technological design of the gambling experience in order to maximize industry profits. Rather than being a symptom of individual addiction deficits, the zone, provided by interactive consumer devices (mobile phones, electronic gambling machines), offers a means to handle the distress of everyday human life (16). One underlying assumption of RG is that consumer protection is best achieved by providing consumers with the information needed to make informed choices about their actions (5, 10). However, the odds of winning or losing, and other statistical probabilities, are seldom transparent when immersed in the game. In the moment, the sensible thing to do is to continue gambling, something that is extensively promoted by advertisements and dreams of “hitting the jackpot” (49). What is considered sensible or rational is, thus, dependent on political values, and the time horizon and the context within which the gambler is located. Throughout our interviews, the ambiguity of wanting to continue to gamble, in the moment, while arguing for stronger coercive measures, is evident. Expecting individuals with gambling problems to take the responsibility for limiting their own access to gambling options and to regulate their behaviors by using RG measures, while in the gambling moment, is thus unreasonable based on the lived experiences of our participants.

In the fifth dilemma, the role of the state is described *via* the tension between emphasizing the need for stricter regulation, to protect vulnerable groups, and the need to preserve individual freedom and avoid violating personal integrity. While the former argument is aligned with a public health approach, the latter argument conforms to the freedom to choose discourse as described by Orford (10). Thus, opinions on state vs. individual responsibility vary, depending on political values and convictions as well as cultural settings (50). The tendency to avoid attributing responsibility to government regulators or gambling providers might be especially strong in certain countries, such as the US (51) and reflects public perception of the government's role vs. the citizen. Absolving the responsibility of state and industry (52) was not as evident throughout this study. This could be understood as related to the extensive gambling problems and subsequent harms of the participants, the recent debate surrounding the roles of regulators and gambling companies within the Swedish gambling market, and the relatively strong public health approach of the Swedish welfare state. Moreover, since the state owned gambling provider Svenska Spel still has a dominant position in the gambling market, gamblers might have higher expectations of the regulating state than in other contexts. What is evident, however, is the struggle portrayed in the gamblers' accounts, where the ideological dilemmas inherent in responsibilities for gambling problems are part of their everyday sense-making. In an analogy comparing gambling with alcohol, a relatively strictly regulated commodity in Sweden (53, 54), the interviewees argue for state responsibility necessitating stronger regulations or even the prohibition of gambling opportunities, advertising, loan markets, and bank transfers. The state should also safeguard funding for care, treatment and research in order to prevent and manage gambling problems. Here, the complex duality emerges between believing that the state should do more or could have done more earlier because they would then have gambled less—at the same time as believing that regulation does not matter because if you want to gamble, you do it regardless.

The participants' approaches toward responsibilities can also be understood in relation to Trnka and Trundle's (4) different domains. The first domain concerns the individual level—the gamblers as self-governing subjects. The participants want to perceive themselves as rational individuals and therefore must claim accountability in relation to themselves, their concerned significant others and their peers in self-help groups and treatment. The second domain concerns the relationship between the gambler, as a customer, and the gambling companies providing potentially harmful products. Critique of the gambling industry's lack of proactivity and compliance with the duty of care, alongside a complete distrust of or disregard for corporate social responsibility as a concept, is pervasive within the participants' accounts. The third domain refers to the relationship between the gambler, as a citizen, and the state responsible for regulating company-activities. The participants, based on their own experiences, argue for a public health approach—with increased responsibility and stronger regulations where the state should be safeguarding vulnerable groups, limiting access to gambling and controlling company-compliance to a greater extent. These domains are situated within certain political contexts and are crucial to understanding the complexity of the different levels that regulation, prevention and treatment of gambling takes place within. These domains, thus, encompass the imposition of conflicting responsibilities on different levels. Where the gambling industry strives for responsibilization of gamblers, the gamblers struggle for recognition and culpability and the state seeks to impose regulation, obliging public health concerns while simultaneously ensuring not to lose too much revenue to private competitors. The participants of this study make use of various, occasionally conflicting, responsibilities on different levels, where values such as sense/sensibility, agent/victim, capacity/incapacity, freedom/control, moral obligations and shame are distinctively actualized and comprehended differently depending on domain. Nevertheless, the participants' critique of the gambling industry's neglect to live up to their claims of adherence to the duty of care, as well as insufficient regulation by the state, is evident.

This study was conducted in Sweden, a country, along with other Nordic countries that has generally had a strong commitment to public health. In the case of gambling, however, the proceeds from the state gambling operator seem to outweigh the harms to the population (55). Moreover, the main revenue (70%) for online or offline Swedish gambling providers of poker, electronic gambling machines and casinos comes from people with gambling problems (30). Despite extensive measures to regulate the gambling market by i.e., restricting bonus offers, introducing a national self-exclusion register and deposit limits (20, 56), the share of people with severe gambling problems has predominantly remained unchanged post-regulation (57). The ultimate responsibility for handling the consequences of gambling remains on the individual, “pathological” gambler. To reduce gambling harm, the Swedish regulator needs to find ways to block payments to unlicensed companies (58) and forcefully sanction providers who neglect to exert duty of care.

As concepts, responsibility and corporate social responsibility can be filled with almost any content depending on political and ideological convictions concerning the role of the state, the gambling market, the freedom of the individual and notions of capacity and intent of various stakeholders. The assumptions that lay the foundation for the responsible gambling discourse are based on the intent and capacity of the individual to make informed choices. But can a person be expected to make rational decisions when risk-taking is encouraged and is essentially the core of the activity, when the probabilities of different consequences of choices are unknown, and when gambler is perceived and handled by society as incapable of making rational decisions? Based on the lived experience of the gamblers in this study, RG's foundational assumptions are irrelevant, erroneous and non-applicable.

### Strengths and limitations

This study includes stories based on lived experiences from gamblers in a context of a reregulation of the gambling market. The data consists of self-reported stories from gamblers with heterogeneous backgrounds and vast experiences of gambling offline or online. Due to the nature of the qualitative data, the results of this study cannot be generalized to gamblers in general. The participants have all experienced extensive negative consequences from gambling, something that should be considered when evaluating the findings. The interviews were conducted during a reregulation of the market, which is why some interviewee's gambling experiences took place previous to the new Gambling Act (20), and why some occurred after. The results are valuable for increasing our understanding on difficulties regarding attempts to reduce excessive gambling habits and the role of RG measures within this process. Furthermore, these results are relevant to consider when developing interventions aimed at reducing gambling harm. Future research should investigate how providers of gambling products in Swedish casinos, EGM venues, gambling shops, and online gambling agents relate to responsibilities and how their role in the responsibilization is actively constructed, in line with what has been studied by Bedford (59) in bingo environments.

## Conclusion

This study illustrates the ideological nature of how responsibilities for gambling problems are comprehended. The participants bear witness of numerous attempts and failures of self-regulation. The dilemma of being responsible while simultaneously incapable constitutes an impossible equation that contributes to substantial feelings of guilt, shame and self-recrimination. The neoliberal discourse, emphasizing individual responsibility, fails to consider the multifaceted dilemmas encountered by individuals every day; the complex motives and vulnerable characteristics of human interactions and behaviors. The gamblers in this study wrestle with making sense of the opposing ideals of responsibility for gambling problems. The ways in which they conduct their lives will depend on how they position themselves within this ideological field. They make use of conflicting discourses, prevalent across society, permeated by power relations and various stakeholder interests on different levels. If individual responsibilization is a prerequisite to be able to reflect on responsibility for gambling problems, self-blame and stigma is unavoidable. The misfit between the RG discourse and gamblers' experiences and needs makes the whole idea of corporate social responsibility irrelevant. To be able to limit extensive gambling harms and design fruitful policy, prevention and treatment measures, the complexity and political implications of the concept of responsibility for gambling problems must be acknowledged and the ultimate burden on the individual eased.

## Data availability statement

The datasets presented in this article are not readily available because due to the nature of this research, participants of this study did not agree for their data to be shared publicly. Questions about the dataset should be directed to ES, eva.samuelsson@socarb.su.se.

## Ethics statement

The study was reviewed and approved by the Regional Ethical Board of Stockholm, 2017/1260-31/5. The patients/participants provided their written informed consent to participate in this study.

## Author contributions

ES designed the study and conducted the interviews and analysis and wrote the first draft of the manuscript. ES and JCÖ discussed the results, contributed to and approved the final manuscript. All authors contributed to the article and approved the submitted version.

## Funding

Funding for this study was provided by the Swedish Research Council for Health, Working Life and Welfare (Forte) within the scope of the Swedish program grant, “Responding to and Reducing Gambling Problems Studies (REGAPS)” (grant no 2016-07091) and the postdoc project, “Dilemmas of Help-seeking—Needs, Experiences and Barriers in Contact with Care in the Case of Gambling and Alcohol Problems” (grant no 2016-00286). Open access publication fees were covered by Stockholm University through the National Library of Sweden (NLS).

## Conflict of interest

The authors declare that the research was conducted in the absence of any commercial or financial relationships that could be construed as a potential conflict of interest.

## Publisher's note

All claims expressed in this article are solely those of the authors and do not necessarily represent those of their affiliated organizations, or those of the publisher, the editors and the reviewers. Any product that may be evaluated in this article, or claim that may be made by its manufacturer, is not guaranteed or endorsed by the publisher.
